# A Plea for Earlier and More Careful Abdominal Diagnosis

**Published:** 1907-02

**Authors:** William Perrin Nicolson

**Affiliations:** Atlanta, Ga.


ATLANTA

### Journal-Record of Medicine

Successor to Atlanta Medical and Surgical Journal, Established 1855, and Southern Medical Record, Established 1870.

OWNED BY THE ATLANTA MEDICAL JOURNAL CO.

Published Monthly.

*Official Organ Fnlton County Medical Society, State Examining Board, Presbyterian Hospital, Etc.*

VOL. VIII. FEBRUARY, 1907. No. 11

BERNARD WOLFF, M. D., EDITOR, 319-320 Prudential Bldg.

M. B. HUTCHINS, M. D., BUSINESS MANAGER, 1015-1016 Century Bld.

E. C. BALLENGER, M. D., ASSOCIATE EDITOR AND ASSISTANT BUSINESS MANAGER,

### ORIGINAL COMMUNICATIONS

### A PLEA FOR EARLIER AND MORE CAREFUL ABDOMINAL DIAGNOSIS.

By William Perrin Nicolson, M.D., Atlanta, Ga.

The great majority of all acute conditions arising in the abdomen, for the relief of which an appeal to surgery becomes necessary, find their beginning under the observation of the general practitioner. It is also true that the succesful treatment of many of these conditions depends, to a great extent, upon their early recognition and prompt handling. So that upon him rests a great responsibility, and it is to this that I would desire to draw attention. Few medical men fail to realize the necessity for physical examination of the chest, but abdominal disturbances are often given over to symptomatic treatment without the same physical examination. The tendency of all of us is to fall more or less into routine, for it is easier to be superficial than thorough, following the law of nature that all forces travel in the line of least resistance. When we go into the subject thoroughly, we depart by force of will from such organic law.

Many grave abdominal affections have a very insidious beginning, and anyone, who is not fully on the alert, is liable to meet with sad surprises, while at the same time the golden opportunity of the patient is lost. The cardinal symptoms, that indicate serious abdominal mischief, are pain, vomiting, and constipation, more or less obstinate, and no one is justified in leaving a patient suffering with either or all of these without a physical examination of the abdomen. A dose of morphine to relieve a colic may mean a loss of many valuable hours to the patient. I am firm in the conviction that adult human beings are not liable to the pains of spasmodic intestinal colic, which belong more certainly to infants and horses. While this statement might be somewhat savagely questioned, I believe that no one will maintain that a pain, continuing after the first opiate has worn off, is spasmodic colic, because true spasm of muscle, once relieved, is cured.

Abdominal pain in the adult of a severe and continued character usually means either appendix or gall bladder trouble, especially when accompanied by vomiting and rise of temperature. Abdominal pain, that continues to be severe for several hours, practically always means one of these. Vomiting, even without pain, and associated with constipation, usually indicates inflammation of the appendix. Perhaps no one factor misleads so many persons as the general distribution of pain to the epigastric, unbilical, of left iliac region, not bearing in mind that pain over the appendix in the first hours of appendicitis is the exception, not the rule. Abdominal pain, confining itself to the epigastrium and radiating to the right shoulder blade, should always call for careful palpation of the region of the gall-bladder. What I would especially urge is that under such circumstances the man, who administers to such patient, without palpating the abdomen at least over the gall-bladder, or appendix, does justice neither to the patient nor himself, and runs the risk of losing for the patient perhaps his only chance of life. It is important to remember that palpation at this time will elicit tenderness over a very circumscribed area, making diagnosis easy, whereas in a few hours the rapid broadening of this area will render accurate diagnosis much more complicated and difficult, and perhaps impossible. Again, it is impossible to elicit the important diagnostic point of abdominal spasm without such physical examination. Should much tenderness be found, let us not fall into the error of telling the patient that he is threatened with appendicitis which is equivalent to one's being threatened with a nail in the foot or a cinder in the eye, and not rest under the quieting suggestion to ourselves that it may be something else, but rather give the patient the benefit of the doubt, and treat it as such, remembering that, as said by Dr. Osler, seventy-five of all acute troubles in the abdomen find their origin in the vermiform appendix.

If I have been somewhat emphatic in my estimate of intestinal colic, what shall I say of the female patient and her ovaries? No man can ever know the number of women, who, being supposed to have suffered from ovarian trouble and peritonitis, now sleep beneath the sod, dead of an appendicitis. It was formerly taught that not over ten per cent. of all cases of appendicitis occurred in women, whereas, I, myself, have operated upon over fifty women suffering with this trouble. Always bear in mind that ovarian pain does not involve the upper region of the abdomen, and that ovarian tenderness does not extend above the iliac spine. General peritonitis in ovarion inflammation is quite rare, and great obstruction of the function of the bowels almost equally so. While ovarian inflammation may secondarily involve the appendix, giving rise to tendernesss over McBurney's point, I think the reverse is often true. Ovarian and tubal inflammation rarely goes to a fatal termination.

Obstinate constipation should demand from the practitioner a careful physical examination of the abdomen. While "obstruction of the bowels" may be in some cases the most feasible diagnosis, it should rarely be so. Every effect must have its cause, and we should use every endeavor to ascertain what it is We should especially look for any local tenderness early in the attack, when abdominal distension has not caused an obliteration of our landmarks. It will be found in a very large number of the so-called "locked bowels," that have swept hundreds to an early grave, have been due to an acute appendicitis. Some time since I saw a boy in consultation with a good practitioner of medicine, whose diagnosis was obstruction of the bowels. He had made no pressure over the appendix, and no physical examination of the abdomen, and, when I did so, and elicited intense pain, he disagreed with my diagnosis of appendicitis, but a few moments afterwards was converted by seeing an immense abscess surrounding a gangrenous appendix. We should never be content with a diagnosis of "obstruction" until we have failed by the most careful examination to eliminate local inflammation at any point.

Turning to the chronic conditions of the abdomen, I would call attention to our frequently sad efforts to do away with a chronic indigestion, especially of an intestinal character, with general nervous disturbance and tendency to colic. Such disturbances of innervation are always due to some cause, the nature of which can be ascertained by careful and painstaking inquiry. I venture to assert that, overshadowing all other causes in importance, are chronic appendicitis of some form or gall-stone. Such patients should be examined carefully, regardles of the absence of the history of previous attacks of either of these troubles, for many patients have been chronic invalids for years from one or both of these conditions without the history of a violent or well-marked attack. I know of no more potent cause of indigestion than an appendix interstitially thickened and intensely hyperesthetic. A patient who is a martyr to dyspepsia, and has an intensely tender appendix or gall-bladder, or both, may be temporarily benefitted by treatment, but to expect to cure him without operation would be as rational as to attempt to cure an eye inflamed from a foreign body with the foreign body still in situ. Here again the ovarian hypothesis comes in with alarming frequency. I recently saw a poor woman, the victim of abdominal pain to the extent of the morphine habit, and chronic invalidism. Though 52 years of age, two prominent gynecologists had said that her only salvation was the removal of her ovaries. I removed an appendix obliterated to a rounded cord, never saw her ovaries, and now she is free from pain, indigestion and opium, and supremely happy and content in perfect restoration to health.

I could cite many cases to illustrate the points that I have raised, but such would simply consume your valuable time. My plea is only for a more careful examination of patients. Let us go to the bottom of things, remembering that it is no more of an affront to a patient to examine the abdomen than to explore the chest. Under the best of circumstances the abdomen is to a certain extent a closed book, and he who deals with its hidden mysteries has many sorrows and disappointments in store for him. None of us can make a diagnosis by inspiration, but we may do so by the cultivation and application of our senses. Do not fall into the fatal mistake of losing the golden chance of reaching a conclusion early instead of being forced to it,
**to surgery to classify the case as surgical, when moribund and medi_**
to surgery to classify the case as surgical, when Moribund, and medical, when it is not, but rather recognize its character early, and be prepared to call assistance before its too late. Rather be like the mariner, who in the color of the sky and the rumble of the distant thunder recognizes and prepares for the approaching storm, than like him, who in the blackening clouds sees no harm, and reefs his sails only when the gale in all its fury has burst above his head. Let us examine our patients more, and not like the Chinese doctor feel the pulse, and examine the patient only when he is in articulo mortis.

